# Respiratory Monitoring by Porphyrin Modified Quartz Crystal Microbalance Sensors

**DOI:** 10.3390/s110101177

**Published:** 2011-01-20

**Authors:** Roman Selyanchyn, Serhiy Korposh, Shunichi Wakamatsu, Seung-Woo Lee

**Affiliations:** 1 Graduate School of Environmental Engineering, The University of Kitakyushu, 1-1 Hibikino, Wakamatsu, Kitakyushu 808-0135, Japan; E-Mails: romanselyanchyn@gmail.com (R.S.); s-korposh@env.kitakyu-u.ac.jp (S.K.); 2 Chitose Technical Center, Nihon Dempa Kogyo Co. Ltd., 1-3-1, Minami-Chitose, Hokkaido 066-0009, Japan; E-Mail: wakamatu@ndk.com

**Keywords:** respiratory monitoring, quartz crystal microbalance, porphyrin based thin films, layer-by layer approach, sensor array

## Abstract

A respiratory monitoring system based on a quartz crystal microbalance (QCM) sensor with a functional film was designed and investigated. Porphyrins 5,10,15,20-tetrakis-(4-sulfophenyl)-21H,23H-porphine (TSPP) and 5,10,15,20-tetrakis-(4-sulfophenyl)-21H, 23H-porphine manganese (III) chloride (MnTSPP) used as sensitive elements were assembled with a poly(diallyldimethyl ammonium chloride) (PDDA). Films were deposited on the QCM resonators using layer-by-layer method in order to develop the sensor. The developed system, in which the sensor response reflects lung movements, was able to track human respiration providing respiratory rate (RR) and respiratory pattern (RP). The sensor system was tested on healthy volunteers to compare RPs and calculate RRs. The operation principle of the proposed system is based on the fast adsorption/desorption behavior of water originated from human breath into the sensor films deposited on the QCM electrode.

## 1. Introduction

Human life activity such as temperature, pulse, respiration and blood pressure can be monitored as vital signs (VS). Assessment of VS is a key routine procedure that is performed during all medical examinations, because it roughly reflects a patients’ status and thus these assessments are repeated often and even persistent monitoring sometimes is required for bedridden patients. Visual observation by medical personnel is the common way to monitor human respiration; however, application of non-invasive respiratory monitoring systems is widespread in routine medical practice [1]. Conventional systems currently used for respiratory measurement tasks include pneumotachography [2], various kinds of pletysmography [3,4], air-filled mattresses [5,6] acoustic noise [7], and capnography as technique for blood carbon dioxide content estimation [8,9]. A set of novel experimental approaches, e.g., fiber-optic sensor based system [10], non-contact ultra-wideband microwave sensor [11], portable magnetometer system [12] and other experimental non-invasive devices [1] can also be found in literature and commercial proposition. Most of the methods give a possibility to assess single respiratory parameter, while ideal monitor of breathing should provide the information about lung ventilation together with some quantitive information about the breath composition [1].

Minute lung ventilation is partitioned into two representative values; tidal volume (*V_t_*) and respiratory rate (RR) [13]. In healthy subjects, RR is approximately 17 breaths/min and *V_t_* is approximately 400 mL. The establishment of a respiration pattern (together with RR and *V_t_*) in normal individuals is a complicated process; however, recognizing alterations in respiratory patterns is important as an early indicator of disease and in many instances this recognition can lead directly to a diagnosis. Therefore, careful observation of RR and respiration pattern is a crucial part of physical examination [14].

Important physical, chemical and biological processes can be followed by observation of the associated mass changes. Quartz crystal microbalances (QCM) are a class of mass-sensitive devices that employ the piezoelectric properties of quartz and have recently been shown as the most widely applied devices. QCMs are thus becoming very popular in the development of sensing systems for application in various fields of human activity. Highly sophisticated automatic, microprocessor-controlled devices that satisfy many scientific and technological requirements are now commercially available [15].

Among the mass sensitive transducers, QCMs are the most utilized, due to their simple preparation and operation according to the well-known Sauerbrey equation [16,17]:

(1) $$\Delta f=-\frac{2f_{0}^{2}}{A\sqrt{\mu_{q}\rho_{q}}}\Delta m$$

where *f_0_* is the resonance frequency of the unloaded QCM, *A* is the active area of the crystal, *μ_q_* and *ρ_q_* are the quartz shear modulus (2.947 g cm^−1^ s^−2^) and density (2.648 g cm^−3^), respectively.

QCM electrodes do not contain any intrinsic selectivity to any chemical species; however, a QCM electrode modified with an appropriate molecule shows high potential for highly sensitive, inexpensive and convenient sensors in medical applications [18]. Efforts over the last decades have resulted in the synthesis of huge variety of synthetic molecular receptors, such as crown ethers, cryptands, spherands, cavitands, porphyrins, calixarenes and cyclodextrins, and these substances can be used for QCM modification to provide a variety of interaction (electrostatic interaction, hydrogen bonding, van der Waals force, and donor-acceptor binding), thus being able to bind cationic, anionic or neutral molecules in a powerful and selective manner [19].

Over the last few decades, porphyrins and their metal containing complexes have been thoroughly investigated as sensitive materials in new sensors development, due to their potential for the development of artificial receptors in chemical sensing [20,21]. Recently, we have employed a layer-by-layer (LbL) process for the preparation of multilayered porphyrin-based films as sensitive elements on optical fibre [22,23] and QCM electrodes [24,25] for the measurement of ammonia gas and relative humidity detection, respectively.

Application of QCMs assembled in a multi-channel sensor array to investigate the chemical composition of breath has been conducted by many research groups [26,27]. Most of the present data in literature is regarding the breath composition aiming the detection of disease biomarkers. To the best of our knowledge, direct dynamical tracking of human respiration by the use of QCM sensors was not reported previously, however similar phenomena was demonstrated by the use of surface acoustic wave sensors [28,29]. In this investigation, we present the approach of respiratory monitoring including RR and RP assessment using surface functionalized QCM sensors. The possibility of on-line measuring of the human respiratory parameters (*RR*, *V_t_*, inspiratory/expiratory flow *etc*.) is demonstrated. Due to the fact that QCM sensors are perfectly and quickly responding to the humidity [24] the indirect measurement of highly humid human respiration is possible. Respiration reflective sensor responses are achieved by pumped delivery of air containing exhaled breath to the sensor chamber. Small and fast changes in humidity during humans inhale/exhale maneuvers are perfectly captured by the porphyrin modified QCM resonators. The proposed system possess several important advantages over conventional methods: sensors are very sensitive so even small alterations in breathing style (RP) can be recorded, system is easy to use, significantly cheaper, compact and robust multi-use and multi-parameter respiratory control systems can be constructed.

## 2. Experimental

### 2.1. Materials

**Figure 1.**
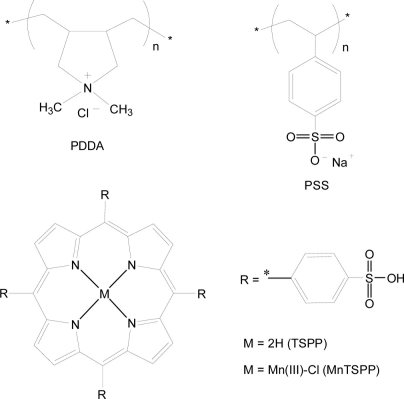
Structural models of the polycation (PDDA), polyanion (PSS), and porphyrins (TPPS and MnTPPS) used for the thin film preparation.

5,10,15,20-Tetrakis-(4-sulfophenyl)-21H,23H-porphine (TSPP, *M*_w_ = 934.99), 5,10,15,20-tetrakis-(4-sulfophenyl)-21H,23H-porphine manganese (III) chloride (MnTSPP *M*_w_ = 1023.38), poly(sodium 4-styrenesulfonate) (PSS, *M*_w_ = 70,000), and poly(diallyldimethyl ammonium chloride) (PDDA, *M*_w_ = 20,000–350,000) were purchased from Tokyo Kasei, Japan. All of these chemicals were guaranteed reagents and used without further purification. Deionized pure water (18.3 MΩ·cm) was obtained by reverse osmosis followed by ion exchange and filtration (Millipore, Direct-Q™). Chemical structures of the porphyrin compounds and polymers are shown in Figure 1.

### 2.2. Layer-by-Layer Film Deposition

The electrostatic LbL method was employed for thin film deposition onto the quartz crystal. The detailed fabrication process, based on the alternate adsorption of oppositely charged polyions, is given in our previous report [24]. Briefly, AT-cut quartz crystals with gold electrodes and a fundamental oscillating frequency of 9 MHz (USI Systems, Fukuoka, Japan) were used as the substrates. Prior to film deposition, the gold-coated electrodes were cleaned and dried. Film assembly was carried out on 2-mercaptoethanesulfonic acid treated QCM resonators. Firstly, the precursor films were deposited by repeating three alternate adsorptions of PDDA and PSS in order to create a planar surface before deposition of the functional coatings. Functional films of 15 cycles (one cycle is a PDDA^+^/TSPP^−^ or PDDA^+^/MnTSPP^−^ bilayer) were prepared by alternate adsorption of PDDA (5 mg mL^−1^ in water) and porphyrin (1 mM in water) onto the quartz crystal. In every case, the alternate film had the porphyrin compound as the outermost layer. The self-assembly process was monitored by measuring the QCM resonance frequency after every deposition cycle.

### 2.3. Experimental Set-Up for Monitoring of Human Respiration

**Figure 2.**
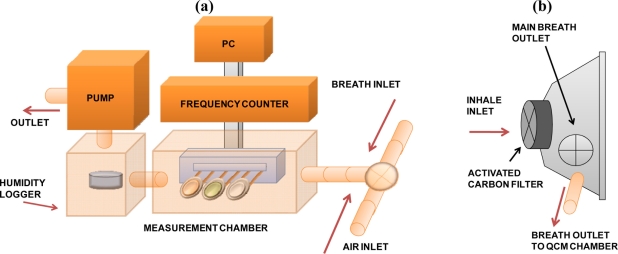
**(a)** Experimental set-up used for breathing monitoring by QCM sensors; **(b)** Schematic illustration of the face mask used for breathing during the experiment.

For the respiration measurements, two QCM sensors coated with the different porphyrin-based films and one additional uncoated electrode were used. A schematic illustration of the experimental set-up used for the respiration signal capture is shown in Figure 2(a). The measurement chamber was connected to a QCM frequency control device (NAPICOS PSA10A, Nihon Dempa Kogyo Co., Ltd., Japan) with a sampling rate equal to 1 sec. A face mask with an inhalation filter (model 3100, 3M, Japan) modified with an additional outlet used for exhaled breath transport to the measurement chamber containing the QCM electrodes [Figure 2(b)] was employed to monitor the breathing oscillation patterns. In respect to the flow inside the chamber the order of electrodes was PDDA^+^/MnTSPP^−^ modified, QCM, PDDA^+^/TSPP^−^ modified and uncoated QCM electrode respectively when listed from inlet to outlet. As additional key components, the following were employed: a mini-pump with regulated flow in range of 0.05–1.5 L min^−1^ (MP-Sigma 30, Sibata, Japan) and humidity logger (DS1923, KN Laboratories, Japan) located in the proximity to QCM electrodes as shown in Figure 2(a). Consequently, the humidity of gaseous mix delivered by pumping can be measured with high precision.

In the proposed measuring setup, the exhaled breath is delivered into the chamber using a pump that provides continuous, uniform and stable breath flow from the mask into the measurement chamber. The use of a pump in our system is very important because it allows reducing the breath humidity by dilution of the exhaled breath with the ambient air and avoids direct condensation of water on the electrodes. Thus, only small portion of the exhaled breath to the QCM containing chamber is delivered at moderate flow (less than 1 L/min), and most of the breath is coming out through the main breath outlet originally present in the mask. During the inhale, the pump is delivering the air which is present inside the mask. The RH and small pressure differences inside the mask induce a specific response of the QCM sensors, which is exactly reflecting human respiration.

The following protocol was used for the monitoring of breathing using the QCM system. Participants were asked to breathe normally in a sitting position while wearing a face mask. Breathing through the nose (usual breathing) was required for 5–10 min while the sensor response was recorded on a computer. For reproducibility check, participants were asked to repeat the breathing procedure three times with rest intervals (*ca*. 5 min) for sensors recovery to the baseline.

## 3. Results and Discussion

The QCM frequency linearly decreased with the increase of the deposited layers, indicating regular growth of the porphyrin-based films [24]. The total frequency change after deposition of 15 cycles for both the (PDDA^+^/TSPP^−^)_15_ and (PDDA^+^/MnTSPP^−^)_15_ films was approximately 2,500 Hz. The frequency shifts for the deposited chemicals correspond to film thicknesses of 43.5 and 46.5 nm for the (PDDA^+^/TSPP^−^)_15_ and (PDDA^+^/MnTSPP^−^)_15_ films, respectively. The detailed information of film deposition process, parameters and characteristics is described in our previous work [24].

### 3.1. Relationship between Sensor Response and Flow Rate

**Figure 3.**
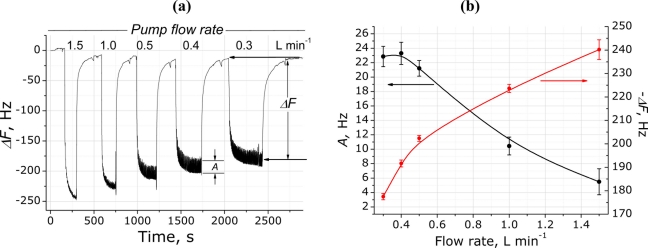
**(a)** QCM sensor response to human respiration when different pump flow rates applied. **(b)** Frequency shift and amplitude of response oscillations due to respiration dependency on the pump flow rate for consecutive breathing episodes of the same person within the same measurement session: open red circles, frequency shifts *ΔF*; closed black circles, averaged amplitude of breath oscillations *A*.

Before investigating the *RR* of different people, the signal dependence on the pump flow rate was assessed. On the example of the (PDDA^+^/TSPP^−^)_15_ film, Figure 3(a) shows a typical QCM sensor response to human breathing when different pump flow rates are applied, which consists of two main features: (i) absolute frequency decrease (Δ*F*) due to the breath delivery into the chamber, which is induced by the increase of humidity and consequent adsorption of water on the QCM electrodes; (ii) amplitude of breath oscillations (*A*) that are attributed to the inhales and exhales and are observed after frequency saturation due to the delivered breath originating RH.

Figure 3(b) shows typical dependencies of both Δ*F* and *A* on the pump flow-rate for the modified QCM electrodes. The averaged Δ*F* decrease with the decrease of the flow rate and this can be explained by the lower volume coming through the QCM chamber and thus fewer molecules are trapped on the electrode surface. In contrast to the Δ*F*, the amplitude of breathing oscillations shows opposite behavior with the decrease in flow rate, namely increasing with the flow rate decrease. This signal change is explained by several factors, including relatively higher changes of flow rates (pressures) in the case of inhalation/exhalation maneuvers, and by longer interaction of breath RH with the sensitive films. Both signals (Δ*F* and *A*) are interesting in terms of their parallel assessment and interpretation; therefore, an optimum flow rate of 0.4 L min^−1^ was selected for further experiments, because good breathing oscillation patterns were obtained for *RR* assessment and also the Δ*F* and *A* values were sufficiently large for further comparison. Additionally, oscillating signal is more stable at lower flow rates (< 0.5 L min^−1^) while at higher than 0.5 L min^−1^ measurement of respiratory signal is quite problematic.

### 3.2. Reproducibility and Breath Humidity Influence

**Figure 4.**
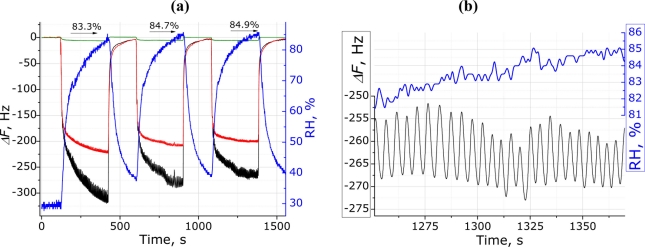
**(a)** QCM sensor reproducibility assessment and humidity influence on all channels response to breathing: green line, blank electrode; red line, (PDDA^+^/MnTSPP^−^)_15_; black line, (PDDA^+^/TSPP^−^)_15_; blue line, humidity change during breathing (right y-axis); **(b)** Enlarged view of the sensor response compared to commercial humidity logger data.

The sensor system showed a good reproducibility for three consecutive respiratory measurement sessions using the blank and porphyrin-modified electrodes (Figure 4). The TSPP modified QCM electrode showed a small difference (*ca*. 25 Hz) in the absolute frequency changes for the three consecutive measurements. For all three consecutive tests, the relative humidity is almost same (mean value 84.3 ± 0.9%), so that its influence cannot explain the decrease in the absolute frequency change. However, the absolute change of relative humidity (ΔRH) at each measurement can be assumed to be a possible reason for such QCM responses. This sensor behavior indicates complex processes occurring on the QCM surface, which are attributed to the interactions between the breath (mainly RH) and the sensitive coatings, together with the flow/pressure influence on the QCM electrode. However, the results presented show a good reproducibility for the signals from the same person. The small change in the frequency shift for reproducible channels could be in a range of the relative measurement error, and therefore, this has no influence on the respiration responsive oscillations and assessment of the respiratory rate in particular. Finally, the response of the unmodified electrode, which was used as reference, was very low (green line in Figure 4), revealing that dynamical adsorption/desorption of water molecules is a major factor of the observed responses.

An additional comparison of the sensor response and humidity logger data is shown in Figure 4(b) (enlarged view). It is evident that it is impossible to track human breath movements recording the humidity data by the commercial device used in this work. However, in contrary to the RH device, due to fast response the surface modified QCM electrodes give an opportunity to track human’s respiration in convenient way.

### 3.3. Signal Differentiation

**Figure 5.**
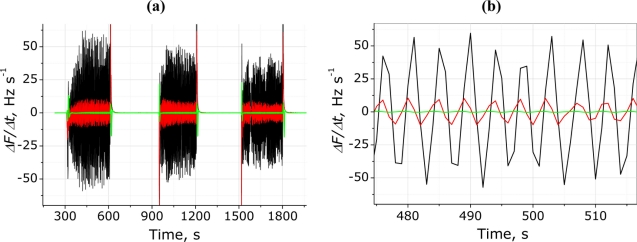
Differential representation of the sensor response used to track breathing movements: **(a)** three consecutive breathing episodes (black, (PDDA^+^/TSPP^−^)_15_; red, (PDDA^+^/MnTSPP^−^)_15_; green, blank); **(b)** enlarged presentation of the differentiated signal.

In order to obtain the common respiratory measurement curve [14,31], the raw QCM signals were differentiated. This simple mathematical operation provides a possibility to obtain a respiration pattern with oscillations around zero level, as shown in Figure 5. Such differential representation of the raw signal is convenient to count RR (maxima or minima per minute). Figure 5(a) shows the differentiated frequency signal respective to the same person during three breathing episodes. The enlarged view in Figure 5(b) shows the differences between the three electrodes, additionally emphasizing the critical role of QCM surface modification, which enable almost two-order amplification of respiratory signal in comparison with one obtained using non-modified electrode.

### 3.4. Interpretation of QCM Response to Human Breathing

**Figure 6.**
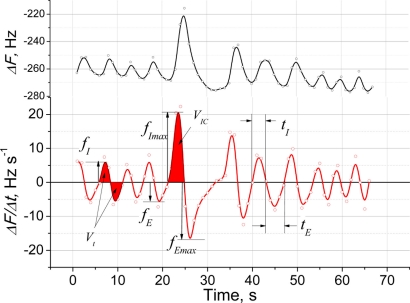
Response of the QCM sensor modified with a (PDDA^+^/TSPP^−^)_15_ film to human breath (upper plot) and the first derivative (bottom plot). Signal features used to characterize the respiratory activity are shown by arrows.

The QCM sensors are sensitive to both exhaled breath and respiratory movements. Figure 6 shows the response of the sensing system to normal human breath with one deep inhalation and forced exhalation maneuver. Interpretation of the signals obtained from the system is important and response features related to the certain useful diagnostic parameters are listed in Table 1. The raw oscillating signal can be used only for RR count, while the differential representation reveals other useful parameters measured in clinical practice [14]. These include tidal volume (*V_t_*) and inspiratory capacity (*V_IC_*), where *V_IC_* indicates a response to the deep inhalation/forced exhalation. The positive and negative maximum peaks extracted from the derivative plot respect to the normal inspiratory and expiratory flows when measured at tidal breathing, and to the peak values (*f_Imax_* and *f_Emax_*) when measured at forced inhale/exhale maneuver. However, all sensor parameters should be calibrated with conventional respiratory measuring systems on further research step. One parameter that can be readily assessed is *RR*, which can be easily counted from the differential graph.

**Figure 7.**
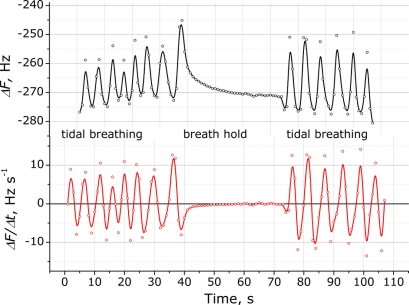
Response of the QCM sensor modified with a (PDDA^+^/TSPP^−^)_15_ film to human respiration (upper plot) and the first derivative of signal to normal human breathing with the breathing hold for approximately 30 s (bottom plot).

To investigate the rapidity of the response the hold of the breathing was measured, when a participant was asked to hold his/her breathing for approximately 30 s. The sensor response shown in Figure 7 demonstrates when the participant’s breathing was stopped. The sensor immediately responded to the stopped breathing within several seconds and this can be observed by the zero value derivative signal approximation. This feature provides one more possible application of the system as a vital (respiratory) activity monitor.

An important advantage of the QCM sensors in comparison with similar approach already presented in literature can be pointed. As shown in works of Rimeika *et al*. [28,29] SAW sensors provide very fast response to humidity (about 1 s to reach the saturation level). However, for the case of human respiratory monitoring this is rather disadvantage. If the saturation level is achieved before the exhale completion, no signal increase will be observed any more and lead to the impossibility of sensor calibration for several respiratory parameters. Since the response of QCM sensors used in the present manuscript is a bit slower, the saturation level is not achieved during the exhalation. So, measurements are made in the kinetic (dynamic) region of adsorption curves. This gives the unambiguous dependence: the higher the measured signal is – the greater breath volume is exhaled.

### 3.5. Respiratory Rate Assessment

**Figure 8.**
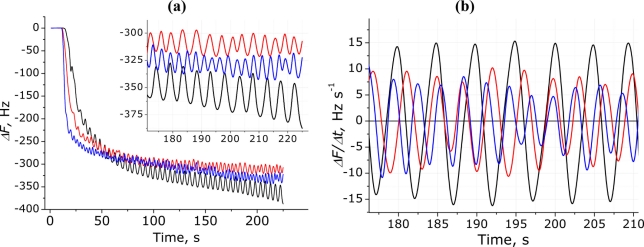
Respiration signals for three different participants obtained with the (PDDA^+^/TSPP^−^)_15_ modified electrode (black line, R; red line, M; blue line, Y): **(a)** absolute frequency change behavior and enlarged RP in inset; **(b)** RP obtained with differentiation of the QCM signal.

Figure 8(a) and inset compare the sensor response for the breathing signals of three different people. The *RR* was counted manually using maximum or minimum values and the following results were obtained: *RR*_R_ = 12, *RR*_M_ = 15, and *RR*_Y_ = 21. One breath movement is considered to be one full signal oscillation (2π).

Figure 8(b) shows differentiated and spline smoothed respiratory signals for the same three participants and allows the observation of significant differences between people; for example, the participant Y has the most frequent breathing with respectively smaller *V_t_*, while the participant R has much deeper and less frequent breathing. However, for all three participants the respiratory signal had the expected RP with clearly indentified inhales and exhales. Differences observed between participants should be linked only to anatomical features and breathing style of certain person.

**Figure 9.**
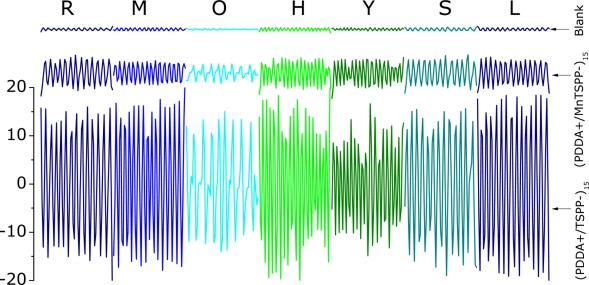
Differential respiratory patterns for seven participants in the *RR* measurement experiment, as responses of two QCM electrodes modified with functional films and uncoated one.

The potential for measurement of *RR* was also confirmed in an experiment with a larger number of participants and the results are presented in Figure 9.

Table 2 summarizes the participant information collected before experiment with the measured *RR* results. The differential signal is significantly different for all samples and only the signals of the participants R, M, S and L appear normal, while the breath signal of the participant O is quite irregular (if examined precisely) with different amplitudes of breathing. It was detected that the participant O had an *RR* of 9 breaths min^−1^, which is below the lower limit or normal range of 12–20 breaths min^−1^ [31], and the participant Y had an increased *RR* of 21 breaths min^−1^; however, there is no clinical significance of these out-of-range values, because all participants were healthy during the experiment and nobody had any health related complaints.

### 3.6. Sensing Mechanism

To explain the sensing mechanism several factors should be accounted. In previous work the same sensitive coatings were used to develop humidity sensors [24] and high RH sensitivity was demonstrated. As the human exhaled breath is highly humid, RH is considered as a main influencing parameter. From this point of view, oscillations of QCM frequency are the response on quickly changed humidity in the sensor chamber when inhale/exhale maneuvers are done. The higher exhaled/inhaled volume—the higher change of humidity occurs and respectively higher signal changes are observed.

Importance of the QCM surface modification becomes obvious when we compare the response of different sensors. The fact of much higher response of (PDDA^+^/TSPP^−^)_15_ in spite of the similar humidity sensitivity [24] highlights the essential difference between measurement of breathing signals and humidity. In the case of humidity or any gas sensing by QCMs, equilibrated steady-state responses are used, while in this work all signals are acquired in conditions of dynamical environmental changes. Thus not only capacity of the film to store adsorbed molecules is important but also desorption/adsorption related properties, *i.e.*, high sensitivity and fast response and recovery time of the sensor devices become a key properties. As a consequence even similar family chemical receptors used in thin films, TSPP containing film provided the best sensing properties in means of high response to the fast changes, taking place when human breathing process used as sample.

Further research is to investigate structural properties of developed coatings, thermodynamic properties, and influence of other factors (e.g., environmental temperature) on sensor performance. Special attention should be devoted to the examination of possible influence of other chemicals present in breath matrix known to contain wide range of compounds present at different concentrations.

## 4. Conclusions

Human respiration was investigated using a QCM three-channel sensor array employing electrodes modified with porphyrin-based functional coatings. Assessment of the useful vital sign parameter, *RR*, was achieved with a simple experimental setup involving two coated and one blank electrodes, while in principle, only one modified QCM electrode was sufficient for *RR* measurement. Additional useful responses, *V_t_* of the lungs, expiratory and inspiratory flows and times, can be simultaneously obtained. *RR* assessment and respiratory patterns were compared for healthy volunteers, and different *RR* values and distinctive patterns were obtained for all participants.

Sensor response accurately reflected the dynamics of human breathing, so that this system could be considered as a novel pneumotachograph based on the mass sensitive principle, which, to best of our knowledge, has not yet been presented in the literature. Moreover, the system could also function as a spirometer, due to ability of recording forced exhalation signals; however, this requires further calibration by conventional approaches.

The presented method is invasive, because mask wearing is required for signal recording, however provides rich respiration related information. In present option this system can be applied for self respiratory assessment, or in critical care and patients under anesthesia, because no additional effort is required to exhale into the chamber. Several important advantages over conventional methods include the high precision due to high sensors sensitivity, so even small alterations in breathing style (RP) can be recorded. System is easy to use and based on the presented principle cheaper, compact and robust multi-use and multi-parameter respiratory control systems can be constructed.

Finally we have demonstrated how porphyrin-based sensitive films assembled on a QCM can be successfully applied in assessment of the human breathing process.

## Figures and Tables

**Table 1.** Assumed relationship between QCM sensor responses and respiratory parameters*.

**QCM Sensor Response**	**Respiratory Parameters [Unit]**
Absolute frequency shift, Δ*F*, Hz	Breath chemical composition including humidity
Frequency of signal oscillations, *RR*, min^−1^	Respiratory rate [min^−1^]
Area under curve, of differentiated signal oscillations, *V_t_*, Hz	Tidal volume [L]
Area under curve, of differentiated signal oscillation at deep inhale, *V_IC_*, Hz	Inspiratory lung capacity [L]
Maxima on derivative signal at normal breathing, *f_I_*, Hz s^−1^	Inspiratory flow [L s^−1^]
Minima on derivative signal at normal breathing, *f_E_*, Hz s^−1^	Expiratory flow [L s^−1^]
Maxima on derivative signal at forced inhale, *f_Imax_*, Hz s^−1^	Peak inspiratory flow [L s^−1^]
Minima on derivative signal at forced exhale, *f_Emax_*, Hz s^−1^	Peak expiratory flow [L s^−1^]
Positive derivative time period, *t_I_*, s	Inspiration time [s]
Negative derivative time period, *t_E_*, s	Expiration time [s]

* Assumption is based on the comparison of the measured QCM signal with typical respiratory pattern [30].

**Table 2.** Data of participants in the *RR* assessment experiment.

**Code**	**Age, y**	**Weight, kg**	**Height, cm**	**Physical activity, h/wk**	***RR* result, breaths min^−1^**
R	28	67	174	2	12
M	21	65	171	7	15
O	21	65	175	0	9
H	20	58	160	8	19
Y	21	65	178	2.5	21
S	28	72	178	7	14
L	42	73	166	4	13
